# Low-penetrance alleles predisposing to sporadic colorectal cancers: a French case-controlled genetic association study

**DOI:** 10.1186/1471-2407-8-326

**Published:** 2008-11-07

**Authors:** Sébastien Küry, Bruno Buecher, Sébastien Robiou-du-Pont, Catherine Scoul, Hélène Colman, Tanguy Le Neel, Claire Le Houérou, Roger Faroux, Jean Ollivry, Bernard Lafraise, Louis-Dominique Chupin, Véronique Sébille, Stéphane Bézieau

**Affiliations:** 1 Service de Génétique Médicale, Pôle de Biologie, CHU de Nantes, 9 quai Moncousu, Nantes, 44093 cedex 1, France; 2 Service de Génétique Oncologique, unité de Génétique Constitutionnelle, Institut Curie, 26 rue d'Ulm, Paris, 75248 cedex 05, France; 3 Laboratoire Biométadys, Université de Nantes, Faculté de Médecine, 1 rue Gaston Veil, Nantes, 44035, France; 4 Biofortis, Nantes, France; 5 Service d'Hématologie, Pôle de Cancérologie, CHU de Nantes, 9 quai Moncousu, Nantes, 44093 cedex 1, France; 6 Service d'Hépato-Gastroentérologie, CHD de La Roche-sur-Yon, Les Oudairies, La Roche-sur-Yon, 85925 cedex 9, France; 7 Association des Gastroentérologues de Vendée, Challans, France; 8 Centre d'Examen de Santé de Saint-Nazaire, France; 9 Centre d'Examen de Santé de La Roche-Sur-Yon, France; 10 Laboratoire de Biomathématiques – Biostatistique, Université de Nantes, Faculté de Pharmacie, 1 rue Gaston Veil, Nantes, 44035, France

## Abstract

**Background:**

Sporadic colorectal cancers (CRC) are multifactorial diseases resulting from the combined effects of numerous genetic, environmental and behavioral risk factors. Genetic association studies have suggested low-penetrance alleles of extremely varied genes to be involved in susceptibility to CRC in Caucasian populations.

**Methods:**

Through a large genetic association study based on 1023 patients with sporadic CRC and 1121 controls, we tested a panel of these low-penetrance alleles to find out whether they could determine "genotypic profiles" at risk for CRC among individuals of the French population. We examined 52 polymorphisms of 35 genes – drawn from inflammation, xenobiotic detoxification, one-carbon, insulin signaling, and DNA repair pathways – for their possible contribution to colorectal carcinogenesis. The risk of cancer associated with these polymorphisms was assessed by calculation of odds ratios (OR) using multivariate analyses and logistic regression.

**Results:**

Whereas all these polymorphisms had previously been found to be associated with CRC risk, especially in Caucasian populations, we were able to replicate the association for only five of them. Three SNPs were shown to increase CRC risk: *PTGS1 *c.639C>A (p.Gly213Gly), *IL8 *c.-352T>A, and *MTHFR *c.1286A>C (p.Ala429Glu). On the contrary, two other SNPs, *PLA2G2A *c.435+230C>T and *PPARG *c.1431C>T (p.His477His), were associated with a decrease in CRC risk. Further analyses highlighted genotypic combinations having a greater predisposing effect on CRC (OR 1.97, 95%CI 1.31–2.97, p = 0.0009) than the allelic variants that were examined separately.

**Conclusion:**

The identification of CRC-predisposing combinations, composed of alleles *PTGS1 *c.639A, *PLA2G2A *c.435+230C, *PPARG *c.1431C, *IL8 *c.-352A, and *MTHFR *c.1286C, highlights the importance of inflammatory processes in susceptibility to sporadic CRC, as well as a possible crosstalk between inflammation and one-carbon pathways.

## Background

As in most Western countries, colorectal cancer (CRC) is a major public health issue in France, where it is the second most common cause of death from cancer among adults [1]. Unfortunately, CRC diagnosis is often made at too late a stage and this induces a dismal prognosis, emphasizing the need for prevention and early diagnostic tools [2]. The development of such tools is, nonetheless, highly dependent on the form of cancer being screened. Thus, reliable genetic tests are already available to detect phenotypically well-characterized familial forms of CRC associated with high-penetrance alleles of genes, including APC in familial adenomatous polyposis (FAP), DNA mismatch repair genes in Lynch syndrome or hereditary non-polyposis colorectal cancer (HNPCC), and MUTYH in MUTYH-associated polyposis (MAP) [3]. However, familial diseases together probably make up only a small percentage of all CRCs [2], and the development of comparable screening tests appears more arduous and remote for sporadic cases, which account for the large majority of CRCs.

In contrast with their major role in familial CRCs, heritable factors are not the only components of the etiology of sporadic CRCs. Susceptibility to sporadic CRCs is multifactorial and derives from multiple interactive combinations of numerous low-penetrance alleles and relevant environmental or behavioral risk factors [2,4]. Independently, each low-penetrance allele contributes modestly to the increase in CRC risk, but its interactions with other susceptibility alleles and environmental factors can lead to a substantial increase in CRC risk, especially when exposed to certain dietary and lifestyle habits [4-6]. The number of interactions is all the higher because the susceptibility genes can be involved in many different biological pathways, which explains the extremely variable phenotype encountered in sporadic CRCs.

Despite much criticism for their non-reproducibility and weak statistical power [6], genetic association studies have been widely used to decypher the mechanisms of cancer susceptibility. Besides, their quality has improved greatly over the past few years [7]. They recently started to produce very valuable results, as illustrated by the identification of several susceptibility loci for colorectal cancers [8-12]. Great expectations can now be held about the results and positive consequences on medical oncology provided by such studies. Beyond the search for susceptibility genes, a global effort is currently being made in the field of sporadic cancers, in order to determine which combinations of genetic variants, i.e., which genetic backgrounds, present at non rare frequencies in the general population, are likely to confer an increase in cancer risk, either alone, or by interacting with usual environmental factors [4,5,13,14].

In order to be part of this effort, we have conducted a case-controlled genetic association study based on a large French population sample. This one already turned out to be very useful by contributing to the finding of the new CRC susceptibility locus at chromosome 8q24.21 made by Zanke et al [9]. Yet, in the present study, our purpose was somehow more modest as we did not attempt to identify new susceptibility loci or variants by a pangenomic approach. Through an exploratory study, we tried to find out whether combinations of variants already known for their possible involvement in carcinogenesis – especially in various Caucasian populations – could determine "genotypic profiles" at risk for CRC among individuals of our French study population. Thus, we focused on 52 allelic variants of 35 candidate genes, selected through a review of the literature on CRC susceptibility, and drawn from five biological pathways relating to inflammation, xenobiotics detoxification, one-carbon, insulin signaling, and DNA repair. Here, we report the results of our investigation on the risk for CRC associated with all 52 allelic variants, analyzed singly or in combinations.

## Methods

### Experimental design and study population

From December 2002 to March 2006, two groups of 1023 patients (632 males and 391 females; mean ± SD age at diagnosis 65.7 ± 10.1 years) and 1121 controls (609 males and 512 females; mean ± SD age at inclusion 61.9 ± 10. years), all of Caucasian origin, were recruited within the Pays de la Loire region of France.

Details of the study population characteristics have been described elsewhere [15]. In short, all cases were patients recruited in regional hospitals and clinics, with a personal history of colorectal cancer diagnosed at an age of ≥ 40 years old. Any patient suspected of having a familial form of colorectal cancer (either Familial Adenomatous Polyposis, Hereditary Non Polyposis Colorectal Cancer or MUTYH-Associated Polyposis) was excluded from the study. Age- and sex-matched controls were recruited at two regional Health Examination Centres; all were ≥ 40 years old and had no family history of colorectal cancer or polyps. Two venous blood samples were collected from each participant in an anonymous manner, after they had legally provided a written informed consent. The study was approved by both the local ethics committee CCPPRB (Consultative Committee for the Protection of Person in Biomedical Research) and by the national French ethics committee CNIL (National Commission for Data protection and the Liberties). Each participant also answered the same one-page standardized questionnaire pertaining to life and food habit information. Endoscopy and histology reports were obtained for each patient.

### Selection of the low-penetrance genes and allelic variants

A review of the literature focused on susceptibility to sporadic colorectal cancer highlighted five biological pathways frequently cited for their role in colorectal carcinogenesis: inflammation, one-carbon, signaling insulin, xenobiotics detoxification, and DNA repair. In these pathways, we selected 35 genes reported as known or possible factors predisposing to CRC (*ALOX5*, *ALOX12*, *IL6*, *IL8*, *PLA2G2A*, *PDL2*, *PTGS1*, *PTGS2*, *PPARG*, *CYP1A2*, *CYP2E1*, *CYP1B1*, *CYP2C9*, *EPHX1*, *GSTA1*, *GSTM1*, *GSTM3*, *GSTP1*, *GSTT1*, *NQO1*, *SULT1A2*, *UGT1A1*, *UGT1A6*, *GH1*, *IGF1*, *IGFBP3*, *IRS1*, *VDR*, *CBS*, *MTHFD1*, *MTHFR*, *MTR*, *MTRR*, *TYMS*, *OGG1*), and chose to investigate 52 polymorphisms -46 single nucleotide polymorphisms (SNPs) and 6 complex polymorphisms – in these 35 genes (Table 1). Among these 52 polymorphisms, 38 polymorphisms were exclusively selected through our review of the literature, because of their possible involvement in the predisposition to CRC [16-30]; most of these polymorphisms reportedly modified the *in vitro *activity of the corresponding gene. It should be noted that 6 of these polymorphisms – found in CYP1A2, CYP2E1, CYP1B1 and CYP2C9-, which were tested previously in our study population [15], are still cited in the present study, given that they were included in the combined analyses described below. The remaining 14 SNPs, located in the candidate genes *PLA2G2A*, *PTGS1*, *PTGS2*, and *PPARG*, were first found by a dHPLC screening in two samples of 50 patients and 50 controls randomly chosen within the whole study population. In fact, we chose to select these 14 SNPs, because of their uneven distribution between the two samples. With the exception of the 4 SNPs in *PLA2G2A*, the other ten SNPs were also found by our bibliographical survey on susceptibility to CRC [5,17,31-34].

**Table 1:** Statistical analysis of association between CRC risk and polymorphisms selected for the study (n = 2144; OR calculations were adjusted according to age and sex).

	**Gene Name**	Nucleotidic change **	Proteic change **	SNP ID **	Ref ***	**Controls**			**Cases**			**+/- vs. +/+**		**-/- vs. +/+**		**+/- and -/- vs. +/+**	
										
						**+/+**	**+/-**	**-/-**	**+/+**	**+/-**	**-/-**	**OR (95%CI)**	**p**	**OR (95% CI)**	**p**	**OR (95% CI)**	**p**
**Inflammation**	ALOX5	c.760G>A	p.Glu254Lys	rs2228065	16	1121	0	0	1023	0	0	Rare		Rare		Rare	
	ALOX12	c.782G>A	p.Gln261Arg	rs1126667	16	361	549	211	357	498	168	0.93 (0.76–1.12)	0.37	0.82 (0.63–1.06)	0.09	0.90 (0.75–1.08)	0.24
	IL6	c.-239G>C		rs1800795	17	435	504	182	363	489	171	1.12 (0.92–1.35)	0.11	1.13 (0.87–1.46)	0.35	1.12 (0.94–1.34)	0.22
	**IL8**	**c.-352T>A**		**rs4073**	17	375	516	230	307	511	205	**1.25 (1.03–1.53)**	**0.02**	**1.12 (0.88–1.44)**	**0.49**	**1.21 (1.01–1.46)**	**0.04**
	PLA2G2A	c.-859C>G		rs11573156	d	738	337	46	627	349	47	1.20 (1.00–1.45)	0.05	1.16 (0.75–1.78)	0.39	1.20 (1.00–1.43)	0.05
	PLA2G2A	c.132C>T	p.Tyr44Tyr	rs4744	d	742	333	46	635	342	46	1.17 (0.97–1.42)	0.05	1.13 (0.73–1.73)	0.47	1.17 (0.98–1.40)	0.09
	PLA2G2A	c.185+88G>A		rs2236772	d	1062	59	0	952	69	2	1.30 (0.90–1.88)	0.14	Rare		1.35 (0.94–1.94)	0.11
	**PLA2G2A**	**c.435+230C>T**		**rs11677**	d	829	276	16	789	226	8	**0.82 (0.67–1.01)**	**0.07**	**0.50 (0.21–1.19)**	**0.13**	**0.80 (0.66–0.98)**	**0.03**
	PLD2	c.1731C>T	p.Thr577Ile	rs1052748	18	316	559	246	280	520	223	1.05 (0.86–1.29)	0.63	1.00 (0.78–1.27)	0.85	1.04 (0.85–1.26)	0.82
	PTGS1	c.22C>T	p.Trp8Arg	rs1236913	d,32,34	976	139	6	879	143	1	1.19 (0.92–1.54)	0.30	0.18 (0.02–1.54)	0.08	1.15 (0.89–1.48)	0.29
	PTGS1	c.50C>T	p.Pro17Leu	rs3842787	d,32,34	970	147	4	874	142	7	1.06 (0.82–1.37)	0.58	2.17 (0.62–7.61)	0.28	1.09 (0.85–1.40)	0.51
	**PTGS1**	**c.639C>A**	**p.Gly213Gly**	**rs5788**	d,32	858	243	20	747	254	22	**1.24 (1.01–1.53)**	**0.03**	**1.26 (0.67–2.36)**	**0.45**	**1.24 (1.02–1.52)**	**0.03**
	PTGS1	c.123G>A	p.Gln41Gln	rs3842788	d,32	1074	47	0	978	45	0	1.10 (0.72–1.68)	0.81	Rare		1.10 (0.72–1.68)	0.81
	PTGS2	c.-646C>T		rs20420	d,16	1121	0	0	1023	0	0	Rare		Rare		Rare	
	PTGS2	c.306G>C	p.Val102Val	rs5277	d,31	783	312	26	707	285	31	1.00 (0.82–1.21)	0.90	1.25 (0.73–2.16)	0.30	1.02 (0.84–1.23)	0.85
	PTGS2	c.1815+427T>C		rs5275	d,31	494	499	128	433	476	114	1.08 (0.90–1.30)	0.36	1.01 (0.76–1.35)	0.91	1.07 (0.90–1.27)	0.47
	PTGS2	c.1815+1912A>G		rs4648298	d,31	1062	59	0	959	64	0	1.31 (0.90–1.89)	0.32	Rare		1.31 (0.90–1.89)	0.32
	PPARG	c.36C>G	p.Pro12Ala	rs1801282	17	896	212	13	822	194	7	1.02 (0.81–1.27)	0.98	0.47 (0.19–1.20)	0.25	0.98 (0.79–1.22)	0.85
	**PPARG**	**c.1431C>T**	**p.His477His**	**rs3856806**	33	875	217	29	803	211	9	**1.07 (0.86–1.33)**	**0.59**	**0.30 (0.14–0.65)**	**0.003**	**0.97 (0.79–1.20)**	**0.8**

**Xenobiotics**	CYP1A2	c.-163A>C		rs762551	19–21	554	482	85	520	423	80	0.97 (0.81–1.16)	0.46	1.08 (0.77–1.51)	0.99	0.99 (0.83–1.17)	0.87
**detoxification**	CYP1A2	c.1548T>C	p.Asn516Asn	rs2470890	20,21	455	525	141	433	467	123	0.97 (0.81–1.17)	0.47	0.97 (0.73–1.28)	0.53	0.97 (0.81–1.16)	0.94
	CYP2E1	c.-1293G>C		rs3813867	20	1032	88	1	954	67	2	0.81 (0.58–1.13)	0.25	2.15 (0.18–25.3)	0.52	0.82 (0.59–1.15)	0.37
	CYP2E1	c.-1053C>T		rs2031920	20	1030	90	1	950	67	6	0.79 (0.57–1.11)	0.20	5.74 (0.67–49.2)	0.09	0.85 (0.61–1.18)	0.33
	CYP1B1	c.1294C>G	p.Leu432Val	rs1056836	19–21	370	577	174	322	510	191	0.98 (0.81–1.20)	0.87	1.22 (0.94–1.58)	0.07	1.04 (0.86–1.25)	0.68
	CYP2C9	c.430C>T	p.Arg144Cys	rs1799853	19–21	823	281	17	751	251	21	0.96 (0.79–1.18)	0.83	1.34 (0.69–2.59)	0.36	0.98 (0.81–1.20)	0.88
	EPHX1	c.337T>C	p.Tyr113His	rs1051740	19–21	564	469	88	525	409	89	0.92 (0.77–1.11)	0.47	1.12 (0.81–1.54)	0.61	0.95 (0.80–1.13)	0.58
	GSTA1	c.-4605G>A		rs3957356	22	372	549	200	333	517	173	1.07 (0.88–1.30)	0.60	1.03 (0.79–1.33)	0.79	1.06 (0.88–1.28)	0.53
	**GSTM1**	**null**			19–21	161	392	568	174	305	544	**0.72 (0.55–0.94)**	**0.01**	**0.86 (0.67–1.10)**	**0.33**	**0.80 (0.63–1.02)**	**0.07**
	GSTM3	c.468+21delAGG			19,20	788	308	25	679	316	27	1.19 (0.98–1.44)	0.07	1.09 (0.62–1.92)	0.42	1.18 (0.98–1.42)	0.08
	GSTP1	c.313A>G	p.Ile105Val	rs1695	19–21	511	486	124	465	447	111	1.04 (0.86–1.24)	0.90	0.96 (0.72–1.29)	0.91	1.02 (0.86–1.21)	0.82
	GSTP1	c.341C>T	p.Ala114Val	rs1138272	19–21	966	146	9	882	137	4	1.00 (0.77–1.29)	0.83	0.44 (0.13–1.47)	0.22	0.96 (0.75–1.24)	0.78
	GSTT1	null			19–21	433	483	205	411	429	183	0.95 (0.78–1.15)	0.49	0.97 (0.75–1.23)	0.62	0.95 (0.80–1.14)	0.58
	NQO1	c.415C>T	p.Arg139Trp	rs4986998	20,21	1056	65	0	961	62	0	1.06 (0.74–1.54)	0.80	Rare		1.06 (0.74–1.54)	0.80
	NQO1	c.559C>T	p.Pro187Ser	rs1800566	19,21	719	351	51	633	343	47	1.09 (0.91–1.32)	0.26	1.08 (0.71–1.64)	0.83	1.09 (0.91–1.31)	0.34
	SULT1A2	c.714A>C	p.Asn235Thr	rs1059491	21	535	458	128	481	437	105	1.09 (0.90–1.31)	0.52	0.92 (0.69–1.23)	0.53	1.05 (0.88–1.25)	0.58
	UGT1A1	c.-3279T>G		rs4124874	23	359	546	216	308	515	200	1.09 (0.90–1.33)	0.34	1.08 (0.84–1.39)	0.54	1.09 (0.90–1.31)	0.37
	UGT1A1	c.-3156G>A		rs10929302	23	535	495	91	474	458	91	1.03 (0.86–1.24)	0.65	1.11 (0.80–1.53)	0.45	1.04 (0.88–1.24)	0.63
	UGT1A6	c.541A>G	p.Thr181Ala	rs2070959	22	508	504	109	470	443	110	0.94 (0.78–1.13)	0.97	1.07 (0.80–1.45)	0.56	0.96 (0.81–1.15)	0.67
	UGT1A6	c.552A>C	p.Arg184Ser	rs1105879	22	460	529	132	438	454	131	0.89 (0.74–1.07)	0.26	1.03 (0.77–1.36)	0.77	0.92 (0.77–1.09)	0.34

**Insulin**	GH1	c.456+90T>A		rs2665802	24	326	561	234	316	527	180	0.96 (0.79–1.17)	0.75	0.80 (0.62–1.03)	0.07	0.91 (0.76–1.10)	0.36
	IGF1	c.-1006CA(19)			25	764	152	205	689	159	175	1.12 (0.87–1.44)	0.24	0.95 (0.75–1.20)	0.63	1.02 (0.85–1.23)	0.81
	IGFBP3	c.-336A>C		rs2854744	25	287	571	263	259	502	262	0.99 (0.80–1.22)	0.80	1.13 (0.88–1.44)	0.42	1.03 (0.85–1.26)	0.76
	IRS1	c.2911G>A	p.Gly971Arg	rs1801278	25	956	158	7	865	152	6	1.01 (0.79–1.29)	0.62	0.86 (0.28–2.66)	0.92	1.00 (0.79–1.28)	0.97
	VDR	c.1024+283G>A		rs1544410	25	423	520	178	372	493	158	1.11 (0.92–1.35)	0.43	1.06 (0.81–1.37)	0.94	1.10 (0.92–1.31)	0.31

**One-carbon**	CBS	c.844ins68			26,27	928	185	8	829	185	8	1.12 (0.89–1.41)	0.32	1.20 (0.44–3.28)	0.82	1.12 (0.90–1.41)	0.31
	MTHFD1	c.1958G>A	p.Arg653Gln	rs2236225	28	339	557	225	322	494	207	0.91 (0.75–1.12)	0.49	0.99 (0.77–1.27)	0.80	0.94 (0.78–1.13)	0.48
	MTHFR	c.665C>T	p.Ala222Val	rs1801133	26,27	457	515	149	435	452	136	0.93 (0.77–1.12)	0.38	0.95 (0.73–1.25)	0.76	0.93 (0.78–1.11)	0.44
	**MTHFR**	**c.1286A>C**	**p.Ala429Glu**	**rs1801131**	26,27	577	443	101	484	432	107	**1.20 (1.00–1.45)**	**0.04**	**1.26 (0.93–1.70)**	**0.12**	**1.21 (1.02–1.44)**	**0.03**
	MTR	c.2756A>G	p.Asp919Gly	rs1805087	26,27	742	335	44	706	289	28	0.90 (0.74–1.09)	0.31	0.64 (0.39–1.06)	0.10	0.87 (0.72–1.05)	0.14
	MTRR	c.66A>G	p.Ile22Met	rs1801394	26,27	322	568	231	291	515	217	1.01 (0.83–1.24)	0.97	1.01 (0.79–1.30)	0.75	1.01 (0.84–1.23)	0.89
	TYMS	c.943+447del TTAAAG			27	524	482	115	456	456	110	1.13 (0.94–1.36)	0.36	1.04 (0.77–1.39)	0.52	1.11 (0.94–1.33)	0.22

DNA repair*	OGG1	c.977C>G	p.Cys326Ser	rs1052133	29	668	402	51	651	321	51	0.84 (0.69–1.01)	0.05	1.04 (0.69–1.57)	0.90	0.86 (0.72–1.03)	0.09

* SNP OGG1 c.977C>G was chosen to be analyzed together with 6 monoallelic germline mutations of the MUTYH gene – also belonging to the base excision repair system like OGG1 – which were studied previously for their predisposing effect on sporadic CRC (Küry et al., Genet Test; ref [30]).

** Nomenclature referring to the databases dbNSP, SNPPER and SNP500Cancer.

*** Ref: numbers correspond to bibliographical references, and the "d" mark corresponds to SNPs identified by dHPLC screening in 50 patients with CRC and 50 controls from the study population.

As regards the nomenclature that we used for the SNPs, we referred to the databases dbNSP, SNPPER and SNP500 Cancer for the ID numbers, and we followed the Human Variation Society (HGVS) mutation nomenclature for the description of nucleotide and proteic sequence variants [35]. Thereby, we corrected wrong notations abundantly used in literature for some of the selected SNPs (e.g., *PPARG *C161T instead of c.1431C>T, or *MTHFR *A1298C instead of c.1286A>C).

### Genotype analysis

Every study participant was genotyped for the 52 polymorphisms selected. Genotypes were determined using high-throughput TaqMan allelic discrimination tests for the 46 SNPs, and fluorescent multiplex PCRs for the 6 complex polymorphisms [for details, see Additional files 1 and 2].

### Statistical analysis

#### Single-SNP association study

All polymorphisms were tested for Hardy-Weinberg equilibrium by a χ^2 ^test in both patients and controls. An appraisal was made on the association between allelic variants and CRC risk, by calculation the odds ratio (OR) of cancer cases together with 95 percent confidence intervals (CI). Association tests consisted of comparing genotype heterogeneity between the groups of patients and controls. For each allelic variant, homozygotes for the major allele were used as the reference group and compared to the other two groups of heterozygotes and homozygotes for the minor allele considered either separately (codominant model), or gathered in a same group (dominant model). We used logistic regression models for assessing single SNP effects adjusted for gender and age covariates. To estimate the risk for CRC, we first performed multivariate analyses based on unconditional logistic regression, adjusting for sex and age at date of reference (date of diagnosis for patients, and date of blood sample collection for controls). To detect a potential overestimation of the odds ratio by unconditional logistic regression, we then employed conditional logistic regression on 811 age- and sex-matched pairs of individuals (75.6% of the whole study population, including 2*324 women, and 2*487 men). In each case, the significance of ORs was assessed by calculation of p-values derived from likelihood-ratio tests.

Multiple testing of 52 polymorphisms implies inevitably a certain rate of false positive results. A correction is therefore required to lower as much as possible the rate of false positives, even though the need to adjust multiple comparisons has been questioned by Rothman [36]. Yet, classical methods of correction such as Bonferroni's adjustment are too drastic for genetic association studies and lead to the rejection of many results, including true associations. We therefore followed the approach proposed by Storey et al., and we estimated the false discovery rate (FDR), more appropriate in large sets of hypotheses than multiple testing procedures [37]. For this estimation, we calculated the q-value, setting the threshold at 0.05. We considered findings that met a false discovery rate (FDR) criterion of 20% to be robust.

#### Multiple-SNP association study

##### Modeling strategy for analysis of genotypic combinations

According to the results of single polymorphisms analyses, we tested all possible combinations of N polymorphisms composed by the N allelic variants found to be significantly associated with CRC risk, and by polymorphisms associated with marginally or non-significant ORs having a p value comprised between 0.05 and 0.25. As a comparison, we also analyzed a hundred of combinations of N polymorphisms randomly selected from any of the 52 ones included in our study. For each polymorphism, the genotype was coded 0 if the rare variant was absent, 1 if present at heterozygous state, 2 if present at homozygous state. Because most of the homozygotes for the minor allele of the polymorphisms tested were often extremely rare, we assumed in our model that the rare effect allele was dominant. In other terms, if a polymorphism predisposed to CRC, genotypes 1 and 2 were considered predisposing to CRC; on the contrary, genotypes 1 and 2 were considered protective if the effect of the minor variant of the polymorphism was rather protective. For each polymorphism included in a combination, we therefore tested two hypotheses: 1. either we considered that the minor allele was predisposing to CRC and therefore the CRC-predisposing genotypes were 1 and 2, whereas genotype 0 was considered protective; 2. or we considered that the minor allele was associated with a decreased CRC risk, and therefore 0 was considered to be the predisposing genotype, whereas genotypes 1 and 2 were considered protective. Thus, for every combination of N SNPs tested, we also tested every possible combination of x protective and N-x predisposing minor alleles (with x ranging from 0 to N), each combination tested representing a different model.

For each test of a combination or model, we compared three groups. The first group was composed by individuals exhibiting genotype 0 for the N SNPs, i.e., carrying the frequent allele at homozygous state for the N SNPs; this group, theoretically the most frequent group in the study cohort, was set as a reference, and the relating combination of genotypes was considered as the "reference pattern" exhibiting a null or very weak effect on CRC risk. The second group was composed of individuals exhibiting patterns of genotypic combinations composed of N predisposing genotypes (predisposing patterns). The third group comprised the individuals exhibiting all other possible combinations of genotypes (mixed patterns).

In the same way as for single SNP association study, heterogeneity between the groups of patients and controls was translated into colorectal cancer risk associated with genotypic patterns, assessed by OR calculation together with 95 percent confidence intervals. We first used unconditional logistic regression, adjusting for sex and age, and we compared the results observed to those obtained by conditional logistic regression analysis restricted to 811 age- and sex-matched pairs of individuals. We eventually assessed the robustness of the associations observed by applying the false discovery rate (FDR) method, setting the threshold at 0.05.

##### Validation of the logistic regression model

In order to validate the logistic regression model, we applied a standard Monte Carlo bootstrap (random resampling with replacement from the original dataset) procedure based on 1000 replicates. We calculated the 5^th ^and 95^th ^percentiles from the resulting distributions to determine the lower and upper limits of the confidence interval.

Alternatively, we examined the internal consistency of the results obtained for the model. Analyses of genotypic combinations were replicated on stratifications of the study population determined according to age, geographical origin, random selection, or inference of population structure [Additional file 1].

## Results

The allele frequencies we found for each of the 52 polymorphisms tested were consistent with those reported in literature and in dbSNP  for Caucasian populations. Two of the 52 polymorphisms analyzed turned out to be monomorphic. The 50 others were all at Hardy-Weinberg equilibrium. Results of the unconditional logistic regression analyses for CRC association with polymorphisms considered independently are described in Table 1. Alternative results of conditional logistic regression restricted to 811 age- and sex-matched pairs of individuals are reported in Additional file 3.

Six associations between CRC risk and allelic variants were determined by both unconditional and conditional logistic regression analyses. For SNPs *PTGS1 *c.639C>A (p.Gly213Gly), *IL8 *c.-352T>A, and *MTHFR *c.1286A>C (p.Ala429Glu), minor alleles appeared associated with an increase in CRC, whereas for SNPs *PLA2G2A *c.435+230C>T, *PPARG *c.1431C>T (p.His477His), they were associated with a decrease in CRC risk. Application of the false discovery rate to the results obtained by unconditional logistic regression analysis showed a value of about 0.5 for these five findings, suggesting that the associations were not very robust. Yet, on the contrary, q-values determined from conditional logistic regression analysis were found between 0.07 and 0.26 for the same findings, rather suggesting quite robust associations [Additional file 3]. Our analyses also showed a protective effect of the variant *GSTM1 *null carried at the heterozygous state on CRC risk. However, this observation is not relevant at the biological level since the loss of activity of the GSTM1 enzyme, or GSTM1 deficiency, is associated with a deletion carried on both alleles. In fact, complementary analyses in our study population revealed that individuals carrying two deleted alleles did not have a significantly different risk of CRC compared to carriers of one or zero deleted alleles (OR 1.08, 95% CI 0.91–1.21, p = 0.4), which is consistent with two meta-analyses performed on this polymorphism [19,38]. Therefore, the CRC-predisposing effect observed for *GSTM1 *null allele is to consider with great caution, all the more because the q-value of 0.4 calculated for this finding would rather suggest a false positive result. Two additional CRC-predisposing effects were observed for SNPs *PLA2G2A *c.-859C>G and *CYP1B1 *c.1294C>G by conditional logistic regression analysis [Additional file 3], but were not found when using unconditional method applied to the whole study population; they were therefore considered as false positives.

Since five SNPs were undoubtfully found associated with a modification of CRC risk in single-SNP analyses, we focused our further analyses on combinations of five allelic variants, as described in Methods. Among all the genotypic combinations tested, only one showed a statistically significant association with an increased risk of CRC, which appeared robust according to the calculation of FDR (q-value < 0.1). This combination was composed by the very same five SNPs that had been found independently associated with CRC risk from single-SNP analyses. Additional file 4 uses the example of this precise set of five SNPs to illustrate the different models of genotypic combinations tested for every set of five polymorphisms – picked from the 52 polymorphisms of the study – which we tested. The most predisposing patterns for this combination, presented in Table 2, combine genotypes 1 and 2 for *PTGS1 *c.639C>A, *IL8 *c.-352T>A, and *MTHFR *c.1286A>C, and 0 for *PLA2G2A *c.435+230C>T and *PPARG *c.1431C>T. These predisposing patterns appeared to be associated with a highly significant increase in colorectal cancer, either compared to the reference pattern alone (OR 2.65; 95% CI 1.58–4.42 with p = 0.0005), or compared to reference and mixed patterns gathered (OR 1.97; 95% CI 1.31–2.97 with p = 0.0009).

**Table 2:** Analysis of association between combinations of genotypes and risk for colorectal cancer, in the entire study population.

**A**. Encoding of theoretical combinations of genotypes					
**Patterns of genotypic combinations**					**Effect on colorectal cancer**
**PTGS1 c.639C>A**	**PLA2G2A c.435+230C>T**	**PPARG c.1431C>T**	**IL8 c.-352T>A**	**MTHFR c.1286A>C**	

0	0	0	0	0	Null or very weak (reference pattern)

0	1 or 2	1 or 2	0	0	Protective (protective patterns) *

1 or 2	0	0	1 or 2	1 or 2	Predisposing (predisposing patterns)
Other genotypes					Average (mixed patterns)

**B**. Analysis of observed combination of genotypes association with colorectal cancer (n = 2144, adjusted by sex and age)					

**Patterns of genotypic combinations**	**Controls**	**Patients**	**OR (95% CI)**	P-value**	

Reference pattern	95 (8.5%)	63 (6.2%)	1.00		
Protective patterns	7 (0.6%)	4 (0.4%)	0.86 (0.24–3.06)	0.8180	
Mixed patterns	978 (87.2%)	890 (87.0%)	1.37 (0.98–1.90)	0.0602	
Predisposing patterns	41 (3.7%)	66 (6.4%)	**2.65 (1.58–4.42)**	**0.0005**	

Reference and mixed patterns	1080 (96.3%)	957 (93.5%)	1.00		
Predisposing patterns	41 (3.7%)	66 (6.5%)	**1.97 (1.31–2.97)**	**0.0009**	

* These patterns were inferred from the identification of the predisposing patterns.

** Observed associations in bold met a false discovery rate criterion of < 0.1.

Validation of the model by bootstrapping and examination of internal consistency strengthened the above results. By bootstrapping, we calculated a mean ± SD OR of 1.86 ± 0.41 (95%CI 1.23–2.85, p = 0.003). Examination of internal consistency confirmed a trend in an increased CRC risk associated with the predisposing combination patterns, regardless of the mode of stratification used [Additional file 5]. No effect of gender or anatomical sub-location was noted. Most of the associations observed were statistically significant, but certain stratifications determined according to geographical origin and/or age displayed a greater effect regarding the predisposing combination patterns (Table 3). Among the 2144 individuals composing the whole study population, the strongest association was found in individuals of ≤ 67 years of age and originating from the French *département *of the Vendée.

**Table 3:** Replications of genotypic combinations analyses on stratifications of the patients and controls groups determined according to geographical origin and/or age.

	**Individuals originating from Vendée**				**Individuals ≤ 67 years**				**Individuals from Vendée ≤ 67 years**			
	
**Genotypic combinations patterns**	**Controls (n = 189)**	**Cases (n = 420)**	**OR (95% CI)**	p*	**Controls (n = 768)**	**Cases (n = 529)**	**OR (95% CI)**	p*	**Controls (n = 134)**	**Cases (n = 232)**	**OR (95% CI)**	p*
Reference pattern	14	29	1.00		65	31	1.00		12	16	1.00	
Mixed patterns	171	359	0.92 (0.47–1.83)		676	460	1.42 (0.91–2.21)		120	195	1.15 (0.52–2.56)	
Predisposing patterns	4	32	**3.80 (1.10–13.19)**	**0.011**	27	38	**3.00 (1.56–5.77)**	**0.003**	2	21	**7.22 (1.38–37.80)**	**0.009**

Reference and mixed patterns	185	388	1.00		741	491	1.00		132	211	1.00	
Predisposing patterns	4	32	**4.14 (1.42–12.08)**	**0.002**	27	38	**2.17 (1.31–3.61)**	**0.003**	2	21	**6.36 (1.44–28.09)**	**0.002**

OR calculations were all adjusted by age and sex.

## Discussion

In this study, we have used a candidate gene approach to examine the associations between colorectal cancer risk and 52 allelic variants distributed in 35 genes drawn from pathways of inflammation, metabolism of xenobiotics detoxification, one-carbon, insulin signaling, and DNA repair. To our knowledge, we are describing here within one of the most comprehensive investigations on populations of this kind, covering more than 1000 patients with sporadic colorectal cancer, and 1000 controls, i.e., in the range of 500–2000 case-control pairs defined by Brennan to detect the statistically significant effect of polymorphisms [39]. Obviously, the list of polymorphisms which we designed here is non-comprehensive, and it must be seen as a panel test, a first attempt in the search for CRC-predisposing "genetic profiles".

With the exception of the 4 SNPs in *PLA2G2A *that we had selected by dHPLC, all the allelic variants chosen for the present study had previously been found to be associated with a modification of CRC risk in at least one study. However, we have been able to replicate the association with CRC risk for only 5 of the 52 polymorphisms tested (Table 1). By independent single-SNP analyses, three SNPs were shown to increase CRC risk: *PTGS1 *c.639C>A, *IL8 *c.-352T>A, and *MTHFR *c.1286A>C. Two other SNPs, *PLA2G2A *c.435+230C>T and *PPARG *c.1431C>T, were on the contrary associated with a decrease in CRC risk. Combinations of the CRC-predisposing alleles relating to these five variants determine "genotypic profiles" at significantly higher risk of CRC (OR 1.97, 95% CI 1.31–2.97). Among the individuals exhibiting these profiles, younger individuals (≤ 67 years) from the Vendée seem to be even more predisposed to CRC (OR 6.36, 95% CI 1.44–28.09). However, the size of the population sample analyzed here is too small to draw definitive conclusions on any age- or geographical-effect. In the same way, the consistency of the risk profile was lost in some of the study population subgroups used for the analyses reported in Additional file 5.b, certainly because of their relatively small size. Caution is therefore required, when considering the true significance of this risk profile, even though bootstrapping results tend to show its robustness [Additional file 5.a]. It is noteworthy that a sixth polymorphism, GSTM1 null allele, showed a protective effect in independent analyses of allelic variants, but the significance of its association with CRC risk remained dubious, and was not confirmed by multiple-SNP analyses, contrary to the five other polymorphisms.

To investigate further the CRC-predisposing combinations that emerged from our analyses, we tested their possible interactions with the environmental and lifestyle risk factors reported in our study questionnaire (physical activity, cooking methods, and consumption of alcohol, tobacco, red meat, cold cuts, white meat and poultry bread, dairy products, fish, fruits, pastries, or vegetables), by use of SNPStats [40]. Indeed, in the same study population, we had previously observed an interaction between allelic combinations of CYP genes and consumption of red meat which leads to a strong increase in CRC risk [15]. In the present study, we did not observe any comparable gene-environment interaction that could remain statistically significant throughout multiple adjustment and/or test for internal consistency in patient and control sub-groups (data not shown). Given that four of the SNPs composing the CRC-predisposing combinations are related to inflammation, the most relevant "environmental" factor to be tested here would have actually been NSAIDs treatment. But, since this item did not figure in our questionnaire on life-habits [15] – we assumed that it would have introduced a bias in the design of the control group-, we were unfortunately unable to analyze its interaction with the CRC-predisposing genotypic combinations of the five SNPs mentioned above.

The respective biological impact of the five variants *PTGS1 *c.639C>A, *IL8 *c.-352T>A, *MTHFR *c.1286A>C, *PLA2G2A *c.435+230C>T, and *PPARG *c.1431C>T provide some clues to the predisposition to CRC associated with some of the genetic combinations that they compose. Thus, *PLA2G2A *c.435+230C>T and *PTGS1 *c.639C>A, belong to genes which encode proteins following each other in the enzymatic cascade of the arachidonic acid pathway. PLA2G2A catalyzes the hydrolysis of membrane phosopholipids, thereby releasing unsaturated fatty acids, including arachidonic acid [41]. The latter becomes the substrate of PTGS1, which in turn catalyzes the formation of prostaglandin H2 (PGH2), a precursor for a number of inflammatory molecules – eicosanoids – that promote colorectal carcinogenesis [42]. Until now, out of the potential key players in the CRC process, more attention has been given to another element of the arachidonic acid pathway, *PTGS2*, rather than to *PTGS1 *or *PLA2G2A *[31,43]. The interest for *PTGS2 *in CRC risk stems from its expression induced by pathophysiological conditions such as tumorigenesis or inflammatory situations [16]. However, we found no significant modification of CRC risk associated with any of the four *PTGS2 *SNPs we tested (Table 1). In contrast, *PTGS1 *is constitutively expressed and its implication in CRC risk has not been investigated very much, since its carcinogenetic role through induction of *PTGS2 *has been suggested only recently [16,34]. Thus, to our knowledge, the synonymous polymorphism c.639C>A (p.Gly213Gly) that we found as having a predisposing effect on CRC (OR 1.24) at the heterozygous (CA) or homozygous state (AA), had not been examined within this context to date and its precise impact on PTGS1 activity would require further functional studies. In the same way, the four *PLA2G2A *SNPs we studied have not yet been tested within the CRC risk context. Therefore, there is no possible point of comparison for the protective effect relating to CRC that we found for the allele c.435+230T carried at homozygous state (OR 0.80, 95%CI 0.66–0.98). Because of its localization within the Mom-1 (Min modifier) locus, and its promotion of tumors in APC^Min ^mice, it had first been suggested that *PLA2G2A *may represent a tumor suppressor gene involved in familial forms of CRC [44]. As a result, such a role of *PLA2G2A *in FAP genesis had been ruled out in humans [41,45]. However, the contribution of *PLA2G2A *to sporadic CRC predisposition cannot be excluded, and it has been suggested that the increase in *PLA2G2A *expression could cause the accumulation of arachidonic acid, a molecule likely to have pro-apoptotic properties [41]. A hypothesis for the protective role of variant c.435+230T might therefore be its enhancement of *PLA2G2A *expression.

*PPARG *c.1431C>T (aka C161T) and *IL8 *c.-352T>A, also relate to inflammation or immune response. PPARγ (peroxisome proliferator-activated receptor gamma) is a nuclear receptor tightly linked to the arachidonic acid pathway, in that it is activated by various eicosanoids [46]. Essential to adipocyte differentiation and to regulation of lipid metabolism, PPARγ is thought to have overall tumor suppressive properties, exerted notably in CRC [46-48]. A protective effect on CRC risk had been reported for the rare allele of variant *PPARG *c.36C>G (p.Pro12Ala) [49,50], but we were not able to reproduce this observation in our study population, perhaps because the reference studies were designed on smaller populations – about 200 case-control pairs-. On the other hand, we noted a significantly decreased risk of CRC associated with the rare allele of variant c.1431C>T (p.His477His) at the homozygous state (OR 0.30, 95%CI 0.14–0.65). These results are consistent with those reported in an earlier study on colorectal adenoma [48], but they are inconsistent with the contrary observations made by another team investigating CRC [33]. Given the controversial effect assigned to *PPARG *c.1431C>T, functional assays would be required to understand the exact role of this little-investigated polymorphism.

As regards *IL8*, it encodes a pro-inflammatory chemokine, released by infiltrating lymphocytes, in response to exposure of the colonic epithelium to toxic and pathologic challenges [50]. The allelic variant *IL8 *c.-352T>A has been found to be associated with CRC risk, even though the effect attributed to rare allele A goes from null [51], to predisposing or even protective [17,52]. As regards our study, we found that genotypes c.352AA or c.352TA were associated with an elevated risk of CRC compared to genotype c.352TT (OR 1.21, 95%CI 1.01–1.46). The debate on the true carcinogenetic role of variant c.-352T>A probably comes from its controversial impact on *IL8 *transcription, sometimes described as an enhancement [51], sometimes as a downregulation [52]. As *IL8 *contributes to chronic inflammation, it would make sense that its overexpression would increase the risk for CRC, and therefore the allele c.-352A may rather enhance the transcriptional activity of the gene.

The fifth gene highlighted by our analyses, *MTHFR *(5,10-methylenetetrahydrofolate reductase), is part of the one-carbon metabolic pathway, involved in both DNA methylation and DNA synthesis. The MTHFR enzyme synthesizes 5-methyltetrahydrofolate, the primary circulatory form of folate, which represents a fundamental methyl donor in cellular metabolism [26]. The influence of MTHFR activity on folate status could be important in CRC neoplasia, since folate deficiency could cause DNA hypomethylation, and/or induce uracil misincorporation during DNA synthesis, which would lead to mutations and chromosomal damage [26,27,53]. Two polymorphisms c.665C>T (also reported as C677T) and c.1286A>C (aka c.1298A>C), have been widely tested for modification of CRC risk, because of the reduction in MTHFR activity induced by their minor allele. In the present study, we found no effect for c.665C>T, whereas we observed an elevated risk of CRC associated with genotypes c.1286CC or c.1286AC compared to genotype c.1286AA (OR 1.21, 95%CI 1.02–1.44). At first sight, these results appear difficult to compare with the greatly inconsistent or even conflicting earlier results, and reviewed in two recent comprehensive meta-analyses [54,55]. However, this discrepancy in the overall results could be explained by the variation of the polymorphisms effect according to folate status. Mu et al. suggested indeed that genotypes reducing MTHFR activity – here c.1286CC or c.1286AC – would favor cancer risk when dietary folate levels are low, by inducing a DNA hypomethylation causing DNA damage and mutations [56]. When folate levels are adequate, the reduced MTHFR activity induced by the same genotypes would lead to a great pool of methylenetetrahydrofolate available for DNA synthesis, and therefore prevent cancer by diminishing uracil misincorporation. Moreover, it has been suggested that, according to its administration prior or further to the existence of preneoplastic lesions, folate would rather prevent or increase tumor development, respectively [53].

All these biological data considered, our work underlines the important contribution of inflammatory processes to CRC susceptibility in our study population, and it points to a possible crosstalk between inflammation and one-carbon pathways. The four allelic variants of genes *PTGS1*, *PLA2G2A*, *PPARG*, and *IL8 *might favor inflammatory processes, whereas the *MTHFR *allelic variant could induce a DNA hypomethylation altering the expression of the putative tumor suppressor gene *PPARG*. Two recent studies on diabetes and cardiovascular diseases have suggested a common pathobiological mechanism between the inflammation process and genotype TT of *MTHFR *c.665C>T (aka C677T), i.e., a genotype inducing a lower MTHFR activity like genotype *MTHFR *c.1286CC [57,58]. However, our results do not enable to conclude to an interaction between the five allelic variants. Indeed, the odd ratios associated with the observed CRC-predisposing combinations are certainly not strong enough, and our method is not the most appropriate one for an investigation on gene-gene interactions.

## Conclusion

Combinations of alleles *PTGS1 *c.639A, *PLA2G2A *c.435+230C, *PPARG *c.1431C, *IL8 *c.-352A, and *MTHFR *c.1286C determine "genotypic profiles" at significantly higher risk of CRC (OR 1.97, 95% CI 1.31–2.97), in a small percentage of the French population. The identification of these CRC-predisposing combinations highlights the importance of inflammatory processes in susceptibility to sporadic CRC, as well as a possible crosstalk between inflammation and one-carbon pathways.

To find their significance, our results would obviously need to be reproduced. Indeed, replication represents a key step for establishing the credibility of any genotype-phenotype association [59]. Only a validation in other Caucasian populations would therefore enable to consider the five above variants and their related genotypic combinations as relevant risk markers for sporadic CRC. Moreover, even though the size of our population sample is quite large, it is not large enough to draw definitive conclusions on the statistical significance of the polymorphisms-disease associations observed. In order to reach such conclusions, our results should be combined to a meta-analysis, since this tool is particularly robust to estimate population-wide effect of genetic risk factors in diseases [60]. In any case, our observations would warrant investigations on possible interactions between NSAIDs and combinations of polymorphisms from the five genes we have highlighted here, which our questionnaire unfortunately did not enable us to achieve. The results of our study also call for more comprehensive investigations into polymorphisms of inflammation genes – especially those belonging to the arachidonic acid pathway-, as candidate risk factors for CRC. In fact, the CRC-predisposing combinations we identified account for only 5% of our study population, which means that many more predisposing combinations, involving different and maybe common polymorphisms, are still to be found. Very large genetic association studies on candidate genes of inflammation may make it possible to find the most frequent predisposing combinations, and thus make the first step towards drawing up recommendations for clinical practice guidelines regarding sporadic CRC.

## Competing interests

The authors declare that they have no competing interests.

## Authors' contributions

SK, BB, and SRdP equally contributed to this work. SK participated in the design of the study, carried out statistical analyses, contributed to genotyping assays, and drafted the manuscript; BB conceived the study, participated in its design, and coordinated the recruitment of patients and controls; SRdP participated in statistical analyses and genotyping assays, and helped to draft the manuscript; CS did samples management and contributed to genotyping assays; HC contributed to molecular genetic analyses; TLN coordinated data storage and management; CLH coordinated the recruitment of patients and controls; RF and JO contributed to the collection of patients samples; BL and LDC contributed to the collection of controls samples; VS participated in statistical analyses; SB conceived the study, participated in its design and coordination and helped to draft the manuscript. All authors read and approved the final manuscript.

## Pre-publication history

The pre-publication history for this paper can be accessed here:



## Supplementary Material

Additional file 1 Supplemental materials and methods. Click here for file

Additional file 2 List of primers and probes used for the study. Click here for file

Additional file 3 Statistical analysis of association between CRC risk and polymorphisms selected for the study using conditional logistic regression (number of age- and sex-matched pairs = 811, i.e., 1622 individuals). Click here for file

Additional file 4 Test of the different possible models of genotypic combinations related to a given set of five polymorphisms picked among the 52 polymorphisms analysed in the study. Click here for file

Additional file 5 Estimation by use of bootstrapping of the external validity of the model showing association between CRC risk and genotypes combinations. Click here for file
