# Supplementary material for: Low-penetrance alleles predisposing to sporadic colorectal cancers: a French case-controlled genetic association study
Source: BMC Cancer. 2008 Nov 7;8:326. doi: 10.1186/1471-2407-8-326 (PMC2585099; doi:10.1186/1471-2407-8-326)
Supplement: Additional file 1 — Supplemental materials and methods. [file 1471-2407-8-326-S1.rtf]

Additional file 1. Supplemental materials and methods.
Genotype analysis
Determination of genotypes by 5' exonuclease assay
Genomic DNA was extracted from collected blood samples using the Nucleon BACC2 kit (GE Healthcare, Uppsala, Sweden). DNA concentrations were calculated using Picogreen technology (Molecular Probes, Eugene, OR) and diluted to 10 ng/ml in 96-well format following a customized automated procedure developed by Microlab Star (Hamilton Robotics, Reno, NV).

Every study participant was genotyped for 46 SNPs of 29 selected genes (Table 1; and Supplemental Table 1). All these SNPs were selected for their relevance to sporadic CRC, according to a review of the literature performed through Pubmed. Genotypes were determined using high-throughput TaqMan allelic discrimination tests. Primers and dye-labeled MGB-NFQ probes were designed and synthesized by Applied Biosystems (Foster City, CA; Supplemental Table 1). Reactions were set up in a 384-well plate in a 6 ml final volume, including: 2.25 ml of 2X Universal Master Mix, 0.11 ml of 40X Assay Mix, 1.64 ml of water, and 20 ng of genomic DNA (10 ng/ml). Reaction plates were thermocycled in an i-cycler (Bio-Rad Laboratories, Hercules, CA): an initial 10 minutes denaturation at 95°C was followed by 45 cycles including a denaturation step at 92°C during 15 s and an annealing/extension step at 60°C during 1 min. After amplification, end-point fluorescence readings were conducted on an Applied Biosystems ABI 7900HT sequence detection system. Genotypes were assigned using the allelic discrimination software SDS v2.1 (Applied Biosystems, Foster City, CA).

Determination of genotypes by fluorescent multiplex PCR
A first multiplex PCR was used to coamplify four complex polymorphisms: a deletion of 3 bp in GSTM3, a deletion of 6 bp in TYMS, a CA repeat in IGF1, and an insertion of 68 bp in CBS (primers sequences are detailed in Supplemental Table 1); reverse primers of the four primer sets used for the multiplex PCR were labelled at their 5' end with FAM. PCR were performed in a final volume of 30 ml including components all provided by the same manufacturer (Eurogentec, Angers, France): 3 ml of 10X mix PCR buffer, 4 mM MgCl2, 200 mM of dNTPs, 0.2 mM of each primer, 1.5 U of HotGoldstar Taq Polymerase and 60 ng of genomic DNA. PCR were performed on a MJ Research PTC-200 (GMI, Minnesota, USA) and consisted of an initial denaturation step at 94°C for 10 mins, followed by 30 cycles of 94°C for 30 s, 64°C for 15 s, and 72°C for 20s, and ended by a 10 mins extension at 72°C. Electrophoresis was performed on a 3130 capillary sequencer, and data were analyzed using the Genescan 3.1 and genotyper 2.5.2 softwares (Applied biosystems, Foster City, USA).

In a second PCR, primers sets were designed to perform a quantitative multiplex PCR of short fluorescent fragments (QMPSF), in order to test whole gene deletion polymorphism of GSTM1 and GSTT1 (Supplemental Table 1). Two primers sets were designed within genes GSTM1 and GSTT1 -adaptating primers designed by Arand et al. [1]-, and a third set of primers was designed to coamplify an additional fragment corresponding to the exon 14 of the MLH1 gene, used as a control as defined by Charbonnier et al. [2]. A universal extension (5'-CGTTAGATAG-3') was added at the 5' end of each forward primer, and another universal extension (5'-GATAGGGTTAG-3') was added to each reverse primer. PCR were performed in a final volume of 30 ml including components all provided by the same manufacturer (Eurogentec, Angers, France): 3 ml of 10X mix PCR buffer, 3mM MgCl2, 200 mM of dNTPs, 0.24 mM of each primer, 1.5 U of HotGoldstar Taq Polymerase and 100 ng of genomic DNA. PCR were performed on a MJ Research PTC-200 (GMI, Minnesota, USA) and consisted of an initial denaturation step at 94°C for 10 mins, followed by 22 cycles of 94°C for 1 min, 60°C for 1 min, and 72°C for 1 min, and ended by a 10 mins extension at 72°C. Electrophoresis was performed on a 3130 capillary sequencer, and data were analyzed using the Genescan 3.1 and genotyper 2.5.2 softwares (Applied biosystems, Foster City, USA). Peak heights of the amplicon obtained from each patient were normalized with the reference gene (MLH1 gene) and then compared with those generated from a normal control. A two-fold reduction in the height of a peak indicated a heterozygous deletion of the gene GSTM1 or GSTT1.

Inference of population structure from genetic data
Stratifications of the cohorts were searched using Structure 2.1 [3], according to the genotype data get for the 52 allelic variants studied. Monomorphic polymorphisms were excluded from the analyses and, following the authors' recommendations to avoid false stratifications, polymorphisms physically too close each others were not included in the analyses. As a consequence, only one allelic variant was analyzed per gene, which led to a global analysis performed on 35 polymorphisms out of 52 (ALOX5 c.760G>A, PLA2G2A c.435+230C>T, PDL2 c.1731C>T, PTGS1 c.639C>A, PTGS2 c.-646C>T, PPARG c.36C>G, ALOX12 c.782G>A, CYP1A2 c.-163A>C, CYP2E1 c.-1053C>T, CYP1B1 c.1294C>G, CYP2C9 c.430C>T, EPHX1 c.337T>C, GSTA1 c.-4605G>A, GSTM1 null, GSTM3 c.468+21delAGG, GSTP1 c.313A>G, GSTT1 null, NQO1 c.559C>T, SULT1A2 c.714A>C, UGT1A1 c.-3156G>A, UGT1A6 c.541A>G, GH1 c.456+90T>A, IGF1 c.-1006CA(19), IGFBP3 c.-336A>C, IL6 c.-237G>C, IL8 c.-352T>A, IRS1 c.2911G>A, VDR c.1024+283G>A, CBS c.844ins68, MTHFD1 c.1958G>A, MTHFR c.1286A>C, MTR c.2756A>G, MTRR c.66A>G, TYMS c.943+447delTTAAAG, OGG1 c.977C>G).
We used both admixture and no admixture models, assuming a number of population K=2 or K=3, and running 100,000  iterations, following a burn-in period of 100,000 iterations.


Figure a. Structure analyses assuming a number of population K=2, and running 100,000  iterations, following a burn-in period of 100,000 iterations. On the left, we used an admixture model. On the right, we used a no admixture model.


Figure b. Structure analyses using a no admixture model, assuming a number of population K=3, and running 100,000  iterations, following a burn-in period of 100,000 iterations.

At least three stratifications were predicted by the software. Analyses of the main effects of the 52 polymorphisms were done, using the three populations as confounder effects. No difference was observed compared to analyses adjusted according to sex and age; the six polymorphisms-CRC associations remained unchanged.
Since the present analysis of stratification was merely exploratory and not confirmatory, these results were not taken into account in the analysis of the main effects. Obviously, the number of polymorphisms included in the present analyses remained too weak to enable a robust stratification, since such analyses would rather require hundreds or even thousands of polymorphisms. However, these stratifications were used as a basis for further analyses on analyses of CRC risk associated with genotyopic combinations, i.e., for the examination of internal consistency for multiple-SNP analyses (Supplemental Table 2). It appeared that the CRC risk observed did not significantly differ from a stratification to another, indicating that the genetic differences between the stratifications determined by Structure were whether artifactual, or did not rely on the five SNPs composing the genotypic combinations studied.

Statistical analysis
Analysis of gene-environment interactions
Analyses of interactions between allelic variants and environmental factors were performed with SNPStats, which proposes statistical models for calculation of risk associated with one pair of interacting variables. For each polymorphism found to be associated independently with modification of CRC risk in univariate analyses, we investigated all the possible interactions with the environmental co-variables of the questionnaire on life habits completed by the study participants. In addition, we calculated 95% CI ORs of cancer cases to determine whether any “polymorphism-environmental factor” pair could modify the risk of CRC in our groups. A likelihood ratio test was used to investigate interaction among variables of interest. The same analyses were performed to assess possible interactions between environmental factors and genotypic combinations of these polymorphisms.
We assumed that there was an interaction between a genetic factor –either a polymorphism or a genotypic combination- and an environmental factor when the effect on CRC risk was two-sided, which means that both factors modified each other's intrinsic effect on CRC risk. For the environmental factor, we compared the associated CRC risk calculated within stratifications of the study population determined according to the genotype with the associated risk calculated in the whole study population, which were reported elsewhere (10). Conversely, for the genotype related to a polymorphism or a genotypic combination, we compared the associated CRC risk calculated within stratifications of the study population determined according to the environmental factor to the associated risk calculated in the whole study population and described in Table 2.
For each positive gene-environment interaction we adjusted the p values obtained for multiple testing, taking into account the twelve environmental risk factors of our questionnaire. Thus, we rejected any results with a p value above 0.001. We also tested the consistency of the observations in sub-groups of the study population determined according to age, geographical origin, random selection, or inference of population structure (Supplemental Data 1).

Supplemental references
1.	Arand M, Muhlbauer R, Hengstler J, Jager E, Fuchs J, Winkler L, Oesch F: A multiplex polymerase chain reaction protocol for the simultaneous analysis of the glutathione S-transferase GSTM1 and GSTT1 polymorphisms. Anal Biochem 1996, 236(1):184-186.
2.	Charbonnier F, Raux G, Wang Q, Drouot N, Cordier F, Limacher JM, Saurin JC, Puisieux A, Olschwang S, Frebourg T: Detection of exon deletions and duplications of the mismatch repair genes in hereditary nonpolyposis colorectal cancer families using multiplex polymerase chain reaction of short fluorescent fragments. Cancer Res 2000, 60(11):2760-2763.
3.	Pritchard JK, Stephens M, Donnelly P: Inference of population structure using multilocus genotype data. Genetics 2000, 155(2):945-959.
